# Supplementary material for: A Pilot Randomized Controlled Trial of a Partial Meal Replacement Preconception Weight Loss Program for Women with Overweight and Obesity
Source: Nutrients. 2021 Sep 15;13(9):3200. doi: 10.3390/nu13093200 (PMC8469733; doi:10.3390/nu13093200)
Supplement: Supplementary file 1 [file nutrients-13-03200-s001.zip › nutrients-1350364-supplementary.pdf]

Supplementary Table S1. Post intervention evaluation data from participants in the two groups.

		Meal replacements ±*n* = 20	Dietary advice ±*n* = 17
Evaluation questions			
**How did you hear about this study?**	*Clinical referral*	6	4
	Facebook	4	1
	Flyer/poster	2	1
	Friend/colleague	2	3
	Internet search	4	6
	Other	2	2
**How did you pick up the advertising for our study?**			
	General Practitioner	8	7
	Social media/colleague	4	2
	University of Sydney	1	0
	Flyers	1	1
	Women&Baby websites	0	1
	Internet search	1	1
	Staff newsletters	1	1
**How would you rate the quality of the support you received?**			
	Excellent	17	12
	Good	3	4
	Poor	0	1
**Did the study meet your expectations?**	*Yes definitely*	14	6
	Yes generally	4	8
	Not really	2	2
	Definitely not	0	1
**Did the weight loss program meet your needs?**			
	Almost all needs met	10	1
	Most of needs met	9	4
	Only a few of needs met	0	8
	None of the needs met	1	4
**Would you recommend the program to a friend:**	*Yes definitely*	12	2
	Yes, I think so	7	5
	No, I don't think so	1	8
	No, definitely not	0	2
**Were you comfortable discussing your weight loss journey?**			
	Very comfortable	18	9
	Mostly comfortable	1	5
	Uncomfortable	0	1
	Very uncomfortable	1	2

**Was the information and resources provided sufficient?**		
……………………………………………….*Totally Agree*	10	3
Agree	8	10
Neither agree or disagree	2	2
Disagree	0	1
Totally disagree	0	1
**How many kilos did you want to lose on the program? 3 to 5**	3	4
5	4	8
5 to 10	7	0
10	5	3
>10	0	1
Don't know	1	1
**Did you change your exercise habits as a result of information received in program?**		
Yes	11	14
No	9	3
**Opinion on the length of the program:** *Adequate*	14	10
Too short	6	6
Too long	0	1
**Adequate number of face-to-face visits:** *Adequate*	15	11
Not enough	5	6
**Could you have completed this program without the face to face visits?**		
**Yes**	3	5
No	17	11
**Usefulness of the online food frequency questionnaire and feedback:**		
Extremely helpful	4	0
Very helpful	11	4
Helpful	0	13
Not that helpful	3	0
Unhelpful	2	0
**Ability to maintain lifestyle changes in the long-term:** *Yes*	9	12
Not sure	10	5
No	1	0

Did you sign-up for the 'Get Healthy' Service?		
	Yes	15
	No	2
How many calls do you receive from the Get Healthy Service?		
	0	1
	1	3
	2	2
	3	4
	5	1
	6	1
	>10	2

Evaluation of the Partial **Meal Replacement** Program

**The virtual consultation on the website was useful**	
Strongly agree	2
Agree	6
Disagree	7
Strongly disagree	5
**Virtual consultation was motivating:** *Agree*	7
Disagree	8
Strongly disagree	5
**Virtual consultation was user friendly:** *Strongly agree*	2
Agree	8
Disagree	7
Strongly disagree	3
**Content on the forums were beneficial:** *Agree*	9
Disagree	9
Strongly disagree	2
**Content on the website was beneficial:** *Strongly agree*	3
Agree	12
Disagree	5
**Were the Classic days with meal replacements, snacks and a high protein meal easy to follow?**	
Strongly agree	3
Agree	14
Disagree	3

**Were the Control days with meal replacements and a vegetable meal easy to follow?**	
Strongly agree	1
Agree	8
Disagree	1
**Did you enjoy the *ad lib* day as part of plan?** *Strongly agree*	10
Agree	10
**Did you enjoy the taste of the meal replacements?**	
Strongly agree	9
Agree	10
Disagree	1
**Were the meal replacements satisfying?** *Strongly agree*	7
Agree	13
**There was enough variety of flavours in the shake range:** *Strongly agree*	7
Agree	11
Disagree	2
**Would you recommend shakes to other people based on taste?**	
Strongly agree	9
Agree	10
Disagree	1
**Did you use recipes from the program recipe book:** *Strongly agree*	3
Agree	7
Disagree	9
Strongly disagree	1
**Did you enjoy recipes from the recipe book:** *Strongly agree*	3
Agree	8
Disagree	9
**Did you feel there was enough content in the guide to understand and follow the program?**	
Strongly agree	4
Agree	14
Disagree	2
**Would you like to continue with the program:** *Strongly agree*	7
Agree	7
Disagree	5
Strongly disagree	1
**Would you prefer face-to-face consultations instead of virtual consultations:** *Strongly agree*	11
Agree	7
Disagree	2

Supplementary Table S2: Changes in dietary intake pre and post intervention

Dietary measure from AES*	Baseline<br>Dietary advice	Meal replacements	14 weeks ±1 month post-program<br>Dietary advice	Meal replacements
% daily energy intake by food group	n= 22	n=25	n=11	n=7
Carbohydrate	42.8 ± 6.7	44.1 ± 6.6	38.8 ± 7.8	47 ± 8.0
Protein**	18.2 ± 3.4	17.6 ± 3.0	21.4 ± 3.4	18.4 ± 4.5
Fats	38 ± 4.1	36.2 ± 4.8	38.1 ± 5.1	33.3 ± 5.9
Saturated fats	14.5 ± 1.9	13.4 ± 2.4	14 ± 2.9	11.1 ± 2.8
Core foods	63 ± 15.1	64.2 ± 13.4	74.7 ± 11.1	79.4 ± 13
Non-core foods	37 ± 15.1	35.8 ± 13.4	25.3 ± 11.1	20.6 ± 13.1
Vegetables	7.4 ± 4.0	10.4 ± 4.8	10.7 ± 5.8	14 ± 5.9
Fruit	5.1 ± 3.8	7.4 ± 5.7	8.4 ± 4.8	14 ± 9.3
Meat	17.4 ± 9.9	13.9 ± 7.6	22.3 ± 8.8	16.3 ± 12.2
Pre-packaged snacks	3.9 ± 2.9	3.4 ± 3.9	2.4 ± 2.2	1.4 ± 2.5
Confectionary	6.9 ± 3.6	6.2 ± 5.0	3.4 ± 3.6	3.9 ± 2.5
Takeaway	10.6 ± 6.6	9.6 ± 5.7	7.1 ± 4.7	5.6 ± 4.6
ARFS ±Australian Recommended Food Score				
	31.8 ± 9	36.4 ± 8.7	36.1 ± 6.3	37 ± 9.9

** difference pre/post significant p = 0.036
